# Fabrication and characterization of electrospun nanofibers using biocompatible polymers for the sustained release of venlafaxine

**DOI:** 10.1038/s41598-022-22878-7

**Published:** 2022-10-27

**Authors:** Heba M. Hashem, Amira Motawea, Ayman H. Kamel, E. M. Abdel Bary, Saad S. M. Hassan

**Affiliations:** 1 Pharmaceutical Analytical Chemistry Department, Faculty of Pharmacy, Mansoura University, Mansoura, 35516 Egypt; 2 Pharmaceutics Department, Faculty of Pharmacy, Mansoura University, Mansoura, 35516 Egypt; 3 Chemistry Department, Faculty of Science, Ain Shams University, Abbasia, 11566 Cairo Egypt; 4 Chemistry Department, College of Science, Bahrain University, Sakhir, 32038 Bahrain; 5 Chemistry Department, Faculty of Science, Mansoura University, Mansoura, 35516 Egypt

**Keywords:** Drug discovery, Chemistry, Materials science

## Abstract

Recently, drug-controlled release nanotechnology has gained special attention in biomedicine. This work focuses on developing novel electrospun polymeric nanofibers (NFs) for buccal delivery of VEN to avoid the hepatic metabolism and enzymatic degradation in the GIT and develop an effective control of drug release. The optimized NFs were obtained by blending polylactic acid (PLA), and poly (ɛ-caprolactone) (PCL) fixed at a ratio of 1:1. It was characterized for morphology, drug-loading, FTIR, XRD, DSC, and in vitro drug release. Ex vivo permeability of the blend NFs was assessed using chicken pouch mucosa compared to VEN suspension, followed by histopathological examination. Further, the cytotoxic effect in three different cell lines using WST-1 assay. SEM morphologies refer to defect-free uniform NFs of PLA, PCL, and PLA/PCL mats. These fibers had a diameter ranging from 200 to 500 nm. The physico-thermal characterization of NFs depicted that the drug was successfully loaded and in an amorphous state in the PLA/PCL NFs. In vitro release of NFs substantiated a bi-phasic profile with an initial burst release of about 30% in the initial 0.5 h and a prolonged cumulative release pattern that reached 80% over 96 h following a non-Fickian diffusion mechanism. Ex vivo permeation emphasizes the major enhancement of the sustained drug release and the noticeable decrease in the permeability of the drug from NFs. Cytotoxicity data found that IC_50_ of VEN alone was 217.55 μg/mL, then VEN-NFs recorded an IC_50_ value of 250.62 μg/mL, and plain NFs showed the lowest toxicity and IC_50_ 440.48 μg/mL in oral epithelial cells (OEC). Histopathology and cell toxicity studies demonstrated the preserved mucosal architecture and the preclinical safety. The developed PLA/PCL NFs can be promising drug carriers to introduce a step-change in improved psychiatric treatment healthcare.

## Introduction

The treatment of central nervous system diseases, including major depression, is evolving in a heightened competitive environment due to the obstacles presented by the blood–brain barrier (BBB)^1^. This barrier prohibits the permeability of therapeutic agents such as large molecules (e.g., proteins or antibodies) and low molecular weight agents, with small molecules possessing lipophilic features being the sole exception^2^. One of the most commonly used medications in the treatment of major depression is Venlafaxine [VEN, (1-[2-(dimethylamino)-1-(4-methoxyphenyl) ethyl] cyclohexanol hydrochloride, Fig. S1a].

VEN is available as an oral formulation and is FDA approved for use in pregnancy as well as in all adult age groups. VEN is well absorbed with a 92% absorption rate from the gastrointestinal tract, and the kidneys excrete 87% within 48 h after ingestion. After absorption, VEN is extensively metabolized in the liver via CYP2D6 predominately into O-desmethylVenlafaxine, ODV^3^, which is its only active metabolite oral administration is generally 2–3 times per day due to its short steady-state elimination half-life of 3–4 h for VEN and ten h for ODV to maintain adequate plasma levels of the drug^4^. The daily dosage of VEN ranges from 75 mg up to a maximum of 375 mg per day. VEN gained widespread clinical use. Oral to both VEN and ODV being potent inhibitors of neuronal serotonin and norepinephrine reuptake and weak inhibitors of neuronal dopamine reuptake.

On the other hand, VEN is also a P-glycoprotein substrate, and P-glycoprotein, a natural barrier to VEN transport, can inhibit VEN penetration into the brain.

Pharmacological activity at the neural serotonin, norepinephrine, and dopamine receptors is believed to be associated with various anticholinergic, sedative, and cardiovascular effects seen with other psychotropic drugs^5^. While generally well tolerated, VEN may cause numerous side effects after oral administration, such as headaches, nausea, drowsiness, constipation, weakness, tachycardia, insomnia, fatigue, and xerostomia.

VEN has extended formulations with high efficacy in decreasing depression symptoms although. Several researchers are working toward finding better techniques to prepare controlled release formulations of VEN^6^. The extended-release formulations have a more impactful effect in improving compliance and enhanced convenience for the patient and a superior risk/benefit ratio compared to the immediate-release formulations of VEN^7^. The commonly used polymers for long-acting medications include pectin^8^, chitosan^9^, and polyvinylpyrrolidone (PVP)^10^. All three have been negatively cited for irritation of the gastric mucosa, uncontrolled drug release, and allergic reactions, respectively. These adverse effects have limited their usage, and none have received FDA approval yet. Some of these polymers are considered suboptimal for future study because of either low drug loading efficiency or not long enough extendibility to cover the period as recommended. It may create a discontinuity between formulation development and a clinical product entering the market^6,7,11–14^. This provides an opportunity to develop novel nano-formulation of VEN based on highly compatible and biodegradable polymers that may achieve the desired results with a different pathway designed to avoid the first-pass metabolism and enhance VEN systemic bioavailability. Under these circumstances, the electrospinning process significantly impacts drug loading efficiency, extended-release time, and integration into nanofibers. Electrospun nanofibers have garnered special interest as a drug delivery system. Owing to their distinct functional features such as eminently minute pore size and enormous surface-to-volume ratio and having a porous matrix that overcomes the limitation of the poor drug payload^15^. Increasing attention has focused on electrospun nano scaffolds as novel materials according to their microporous architecture, the physicochemical properties of the utilized polymers, and modulating the formulation variables That offer ample flexibility to customize the drug release behaviors^15^. Their bioactivity, resorbability, biocompatibility, and degradability provide significant benefits in drug delivery systems.

Polylactic acid (PLA, Fig. S1b) is widely used in biomedical applications and is produced from renewable sources^16,17^. PLA is either a semi-crystalline or amorphous biopolymer according to its relative ratio of PLA backbone chains synthesized in the chemical process. Its biodegradable products are highly secure in the human body and considered appropriate hydrophobic media for effective hydrophilic or hydrophobic drug loading^18^. Poly (ɛ-caprolactone) (PCL, Fig. S1c) is another synthetic semicrystalline polymer commonly utilized in medical applications. PCL has good drug permeability, a slow degradation rate, and extraordinary physicochemical properties for chemical modifications^19^. The synergetic effect of PLA/PCL blended nanofibers results from 3D nanomaterials that have a shorter time of degradation, higher mechanical features, and improve the bioactivity of the drug. The PLA /PCL blend has impactable features and benefits of the physicochemical properties of biopolymeric materials different from those of the individual virgin biopolymers^20,21^. The blend formation is simple and easier to become spinnable and therefore capable of achieving the enhanced material properties of a suitable blend of individual biopolymeric components^22^. These biopolymeric composite nanofibers have the potential to be used for drugs that require a high drug concentration in the oral cavity for sufficient drug absorption through the buccal mucosa^23^. The buccal route of drug administration is currently in use for the treatment of opioid use disorders and can potentially be a life-saving option in treating a patient’s unconscious from an uncontrolled seizure^24^.

Although the controlled delivery system for VEN has already been reported in the literature^6,8,11–14,25–27^, no report is available on using VEN-PLA/PCL nanofibers as a vehicle for the extended buccal release of the drug. This study was developed to inspect the effect of innovative FDA-approved biodegradable and biocompatible VEN-PLA/PCL NFs as a vast delivery system for the buccal administration of VEN.

## Methods

### Materials

Venlafaxine hydrochloride (VEN) was kindly supplied as a gift sample from Pharaonia Pharmaceuticals (Alexandria, Egypt). Polylactic acid (PLA) (Mw 93,500 g/mol) was purchased from NatureWorks (Minnetonka, United States). Poly (ɛ-caprolactone) (PCL) (Mw 80,000 g/mol), Chloroform (CFM), and dimethylformamide (DMF) were purchased from Sigma-Aldrich (Steinheim, Germany). Liver cancer cells (Huh-7), Oral epithelial cells (OEC), and green monkey kidney cells (Vero) were garnered from Biomedical Research Section, Nawah Scientific Inc. (Mokatam, Cairo, Egypt). All other chemicals and solvents were of analytical grade and utilized as purchased.

### Preparation of the composite NFs

A series of solutions with varied biodegradable polymer concentrations was prepared as listed in Table 1. A 10% (w/v) PLA solution was prepared by dissolving the proper amount of biopolymer in CFM/DMF binary solvent (80:20 v/v). Meanwhile, two different solutions of PCL (10% and 14% w/v) were prepared in binary solvent (70:30 v/v). The solution of a combined biodegradable polymer composite of PLA and PCL at a 1:1 volume ratio was prepared in binary solvent (70:30 v/v). Based on preliminary studies, the fixed amount of VEN (20% w/v) was dissolved in the biopolymer solutions. All solutions were magnetically stirred for three h at room temperature using a magnetic stirring Hot Plate (Hot Plate and Magnetic stirrer, Daihan Scientific Co., LTD, Korea). Plain NFs, as a control, were obtained with identical compositions without adding VEN. Meanwhile, cast films of the utilized biopolymers were obtained for comparative study with similar quantitative compositions of NFs.

**Table 1:** Composition of different nanofibers.

Formulation	Polymer	Polymer concentration (%)	Polymer ratio (wt/wt)	Solvent ratio (v/v) (CFM: DMF)	Flow rate (mL/h)
F1	PLA	10	–	80:20	1.0
F2	PCL	14	–	70:30	0.6
F3	PLA:PCL	10	50:50	70:30	0.3

Electrospinning was carefully performed using NANON-01A electrospinner (MECC CO., LTD., Japan) combined with a high-voltage power supply, an infusion pump, a glass syringe attached to a stainless steel-smooth needle (outer diameter, 1.2 mm; inner diameter, 1.0 mm), and drum stainless steel collector. All prepared solutions were ejected at a constant voltage of 20 kV between the syringe needle and drum metal collector during the electrospinning process. The PLA, PCL, and PLA/PCL blend solutions were sprayed at 1.0, 0.6, and 0.3 mL/h flow rates, respectively, with a constant air gap of 15 cm, straggled the needle tip and the collector. To fabricate the nonwoven and homogenous thick mesh, all resultant fibers were collected (diameter,40 mm; rotating speed, 200 rpm of a drum) on a flat aluminum foil sheet covering a drum collector. The electrospinning process was executed at 24 °C and with low humidity. The collected fibers were dried under a vacuum to eliminate the residual solvents and all moisture completely.

### Characterization techniques

#### Scanning electron microscope

The microscopic morphologies and size of the cast films and NFs were examined using an SEM (JEOL JSM-6510LV, Oxford Instruments, UK) at an acceleration voltage of 30 kV. The average diameter of the fibers was calculated using an image analysis tool in the Zeiss smart SEM® software for their quantification. Before the examination, the fibers were attached to an aluminum stub with double-sided adhesive tape and then sputter-coated with gold before imaging to render them electrically conductive.

#### Attenuated total reflectance-Fourier infrared spectroscopy

Infrared spectra of the drug, polymers, and electrospun NFs were recorded on the FTIR spectra. This was fitted with an attenuated total reflectance mode (ATR) (Thermo-Fisher Scientific, Inc., Waltham, MA, USA) and a single-reflection diamond crystal using a Nicolet iS10 spectrometer. The resulting spectra were investigated over the 4000 to 550 cm^− 1^ with a resolution set at 4 cm^− 1^.

#### X-ray diffraction

The crystallinity of the pure drug and the formulations were inspected using XRD (Shimadzu XRD-6000 diffractometer, Japan). The XRD was operated at 40 kV and 40 mA using Cu-Kα radiation in the range of (2θ) 5°–55° with a scanning rate of 0.05°/s at room temperature using the monochromatized diffractometer (λ = 0.154 Å) with graphite-sample monochromators. The crystallinity level was detected by applying the area integration method^25^.

#### Differential scanning calorimetry

The thermal features of VEN, polymers, and the NFs formulations were recorded using DSC (SII DSC 6200, Japan). Sealed samples were heated in aluminum pans, and an empty pan was used as a reference. In a nitrogen atmosphere of 20 mL/ min flow rate, thermograms were recorded in the temperature range of 50 °C–400 °C with a heating rate of 10 °C/min.

#### Evaluation of drug encapsulation efficiency

The drug entrapment efficiencies of the nanofiber mats were calculated as follows. 10 mg of dried drug-loaded nanofibers were cut and dissolved in 10 mL CFM for 5 min using a magnetic stirrer. The amount of the drug was analyzed by a UV/VIS spectrophotometer (Shimadzu UV-1601 PC, Japan) at a wavelength of 285 nm and quantified using a previously constructed standard curve. The test was conducted in triplicate, and the results were calculated as mean ± SD. The drug entrapment efficiency (EE %) of the process was evaluated using the following Eq. ^28,29^:

1 $$ {\mathbf{EE}}\% \, = \frac{Amount\,of\, drug\, in\, NFs}{{Drug\, initially\, added}}\;{\mathbf{X}} \, {\mathbf{100}} $$

#### In vitro drug release

In vitro release of VEN from the selected nonwoven nanofiber mats was studied using dialysis bags. NFs and cast films were divided into pieces holding 25 mg of VEN, and each was placed into 50 mL of PBS (phosphate-buffered saline, pH 7.4) and then underwent a rotary shaker incubation (GFL Gesellschaft für Labortechnik, Burgwedel, Germany) at 100 rpm at 37 °C ± 0.5 °C. Samples were withdrawn and reconstituted with the dissolution medium at predetermined intervals (0.5, 1, 1.5, 2, 3, 4, 6, 24, 48, 72, and 96 h). The collected samples were filtered through a 0.45 μm Teflon syringe filter and assayed spectrophotometrically at 226 nm for drug content. All incubation processes were performed in triplicate, and the results were expressed in terms of cumulative release as a function of time according to the following Eq. ^30^.

2 $$ {\mathbf{The}} \, {\mathbf{cumulative}} \, {\mathbf{drug}} \, {\mathbf{release}} \, \left( \% \right) \, = \frac{Ct}{{C\infty }} \times 100 $$

where C_t_ is the amount of VEN released after time t and C_∞_ refers to the total amount of drug loaded theoretically into the NFs.

#### Mathematical models for drug release kinetics

To explain the mechanism of VEN release, release data were kinetically analyzed according to zero-order, first-order^31,32^, and Higuchi diffusion mechanism^33^. For further investigation of release mechanisms, the Korsmeyer-Peppas model was applied to determine the diffusion exponent (n)^34^. The model to which the release data were best fitted with the highest correlation coefficient (R^2^) was the one that was utilized to calculate the release of VEN.

### Ex vivo assessment of buccal delivery systems

#### Ex vivo drug permeability test

The optimized formulation, with promising in vitro results, was exposed to drug permeation testing using chicken cyst tissues attached to modified Franz diffusion cells.

Chicken cyst tissues were selected as a model membrane for the permeability study due to their histological similarity with buccal mucosa^35^. Chicken cyst tissues were freshly collected post-sacrifice from a local slaughterhouse. The tissues were firstly pre-treated by cleansing the loose connective fibers. Surface fats were eliminated, and the tissues were then cleansed with isotonic phosphate buffer (IPB) pH 7.4.

The washed tissues were instantaneously utilized within which their surface facing up, and the donor compartments contained 2 mL of simulated saliva^36^. All receptor compartments were filled with IPB pH 7.4, and the membrane was placed just below the surface of the recipient solution. All diffusion cells were placed in the rotary shaker with a temperature set at 37 °C and at 100 rpm. The samples were withdrawn at predetermined time intervals over 6 h, filtered through a 0.45 µm Teflon syringe filter, and reconstituted with an equal volume of fresh IPB. The results were recorded using spectrophotometric measurements at 226 nm. The tests were conducted in triplicate (n = 3), and the average flux and permeability coefficient values were calculated^37^.

The cumulative percentage of permeated drug per square centimeter was plotted versus time (h). The steady-state flux was calculated from the slope of the linear fraction of the plot as in the following equation:

3 $$ {\mathbf{Flux}} \, = \, {\mathbf{Jss}} \, = \frac{{ \frac{dQ}{{dt}}}}{A} $$

where *Jss* is the steady-state flux; d_Q_/dt is the permeation rate; A is the diffusion surface area (3.46 cm^2^).

The permeability coefficient *P* was calculated using the following equation:

4 $$ P = \frac{Jss}{{Cd}} $$

where *P* is the permeability coefficient and C_d_ is the initial drug amount on the donor side.

#### Histological examination

At the end of the permeability study,the chicken cyst tissues were histologically examined to investigate mucosal changes, such as damage or irritation^35^. After carefully removing the pouch mucosa from the diffusion cells, it was washed with IPB to remove any residual dosage forms. The tissues were fixed with 10% formalin and mounted in hard paraffin. They were successively partitioned and microscopically inspected after smearing with hematoxylin and eosin. Cellular stealth and tissue destruction were detected with a light microscope^38^.

#### In vitro study of cell toxicity

The cytotoxic effects of pure VEN, plain NFs, and medicated NFs (F_3_) were studied in vitro using liver cancers cells (Huh-7), OEC, and green monkey kidney cells (Vero), which were all garnered from Biomedical Research Section, Nawah Scientific Inc., (Mokatam, Cairo, Egypt). This work was approved by the Ethical Research Committee of the Faculty of Pharmacy, Mansoura University. Briefly, the cells were cultured in Dulbecco's modified eagle medium complemented with streptomycin (100 mg/mL), penicillin (100 units/mL), and 10% of heat-inactivated fetal bovine serum in a humidified atmosphere of 5% (v/v) CO_2_ at 37 °C. Cell viability was assessed by WST-1 (Water Soluble Tetrazolium Salts) assay. Aliquots of 50 μL cell suspension (3 × 10^3^ cells) were sown in 96-well plates and brooded in complete media for 24 h. All samples were sterilized by UV irradiation before use. Afterward, the cells were exposed to another aliquot of 50 µL media containing pure VEN at concentrations of 0.01, 0.1, 1, 10, and 100 µg/mL. VEN-equivalent amounts of plain and medicated NFs were used to prepare the above concentration series. After 48 h of exposure, the cells were exposed to a 10 μL WST-1 reagent. The absorbance was detected after 1 h at 450 nm (A_450_) using a BMG Labtech®- Fluostar Omega microplate reader (Allmendgrün, Ortenberg, Germany). All of the experiments were performed in triplicate. Representative phase-contrast images of cells were captured just before adding the WST-1 reagent (Olympus BX 41 microscope, Olympus, USA). The cytotoxic effect was expressed as the percent cell viability and was used to construct a dose–response graph. The half-maximal inhibitory concentration (IC_50_, the concentration that killed 50% of cells) compared with the untreated (control) cells was calculated from the dose–response curve. The percent cell viability was measured using the following equation.

5 $$ Cell\, Viability \% = \frac{{A_{450}\, of \,treated\, cells}}{{A_{450}\, of\, control\, cells}} \times 100 $$

#### Statistical analysis

The EE%, in vitro, and ex vivo experiments were all performed, and the data are reported as mean ± SD of at least three replicates. The results obtained were compared using a one-way analysis of variance followed by Tukey–Kramer multiple comparisons. An unpaired student t-test was used to compare the means of the ex vivo permeation study, with the ρ-value set as 0.05.

#### Results

### Characterization of NFs

#### Electrospinning conditions and morphology of various nanofibers

**Figure 1:**
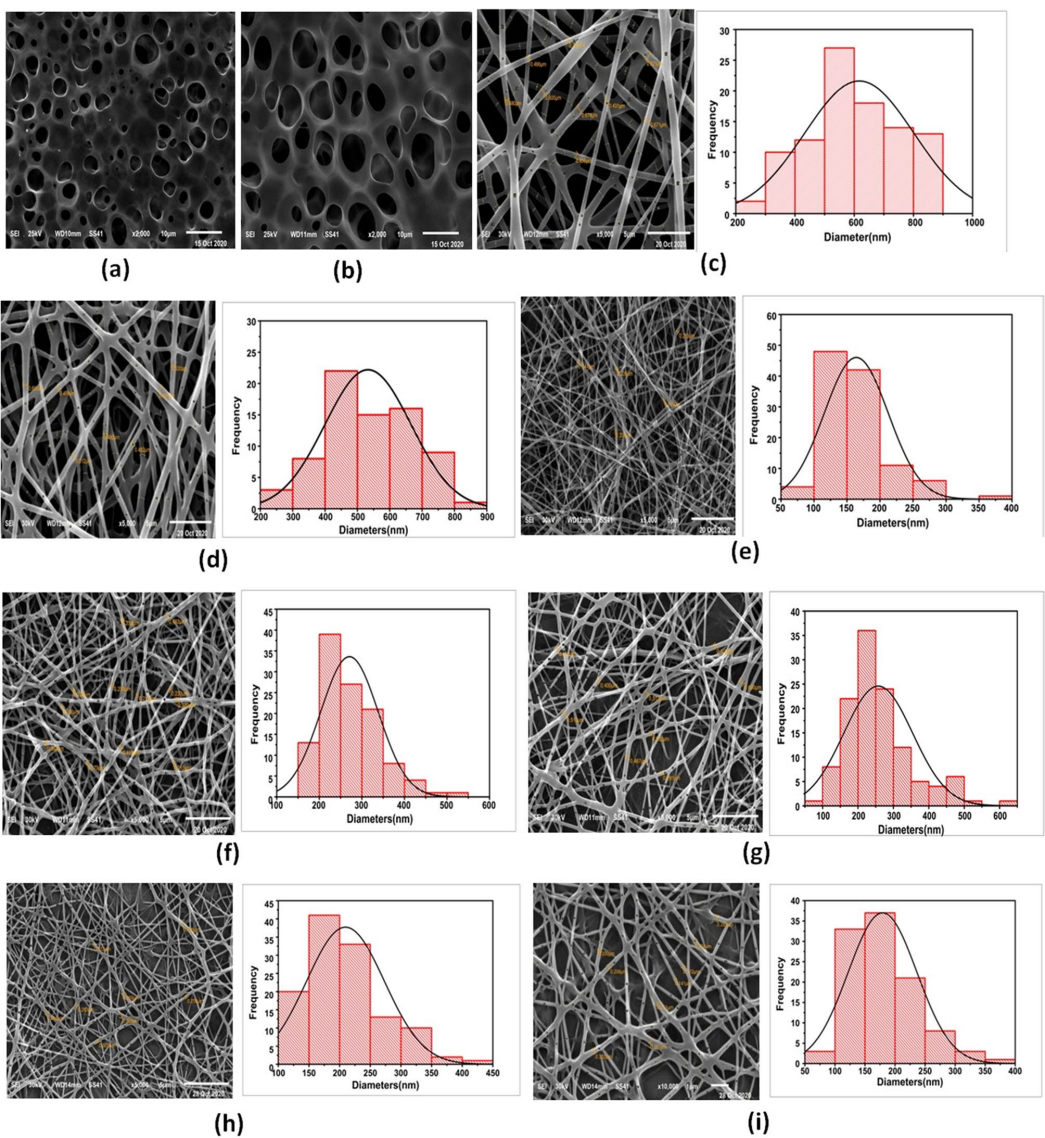
SEM micrographs of (**a**) PLA cast film, (**b**) VEN-PLA cast film, (**c**) 10% PLA NFs, (**d**) VEN-10% PLA NFs (F_1_), (**e**) 10% PCL NFs, (**f**) 14% PCL NFs, (**g**) VEN-14% PCL NFs(F_2_) (**h**) 10% PLA/PCL composite NFs, and (**i**) VEN-10% PLA/PCL composite NFs (F_3_).

Figure 1 represents the morphology and sizes of all the different prepared samples of the cast films and NFs mats (F_1_, F_2_, and F_3_) as obtained from SEM examination. Figure 1a and b show smooth fracture surfaces of plain and medicated PLA cast films, respectively. After the electrospinning process, Fig. 1c and d show the smooth defect-free NFs with a narrow diameter distribution and an average size of about 500 nm designated as “plain” and the medicated NFs of PLA, respectively. On the other hand, to optimize electrospinning conditions in order to fabricate well-defined nanofibers of PCL polymer, the concentration of PCL varied from 10 to 14 wt%. As seen in Fig. 1e, 10% of PCL NFs exhibit very narrowly, smooth, and beads-free NFs with average diameters ranging from 150 to 250 nm. The average diameter of 14% PCL NFs was increased to a predominant size of 300 nm owing to increasing PCL concentration from 10 to 14 wt% as demonstrated in Fig. 1f and g. Pure PCL NFs appeared smaller and much finer than the pure PLA embedded NFs and the same applies to both drug-incorporated PCL and PLA NFs^39^. Alternatively, PLA/PCL blended NFs produced smooth, very narrow, and uniform electrospun fibrous structures as seen in Fig. 1h and i.

#### ATR-FTIR analysis

**Figure 2:**
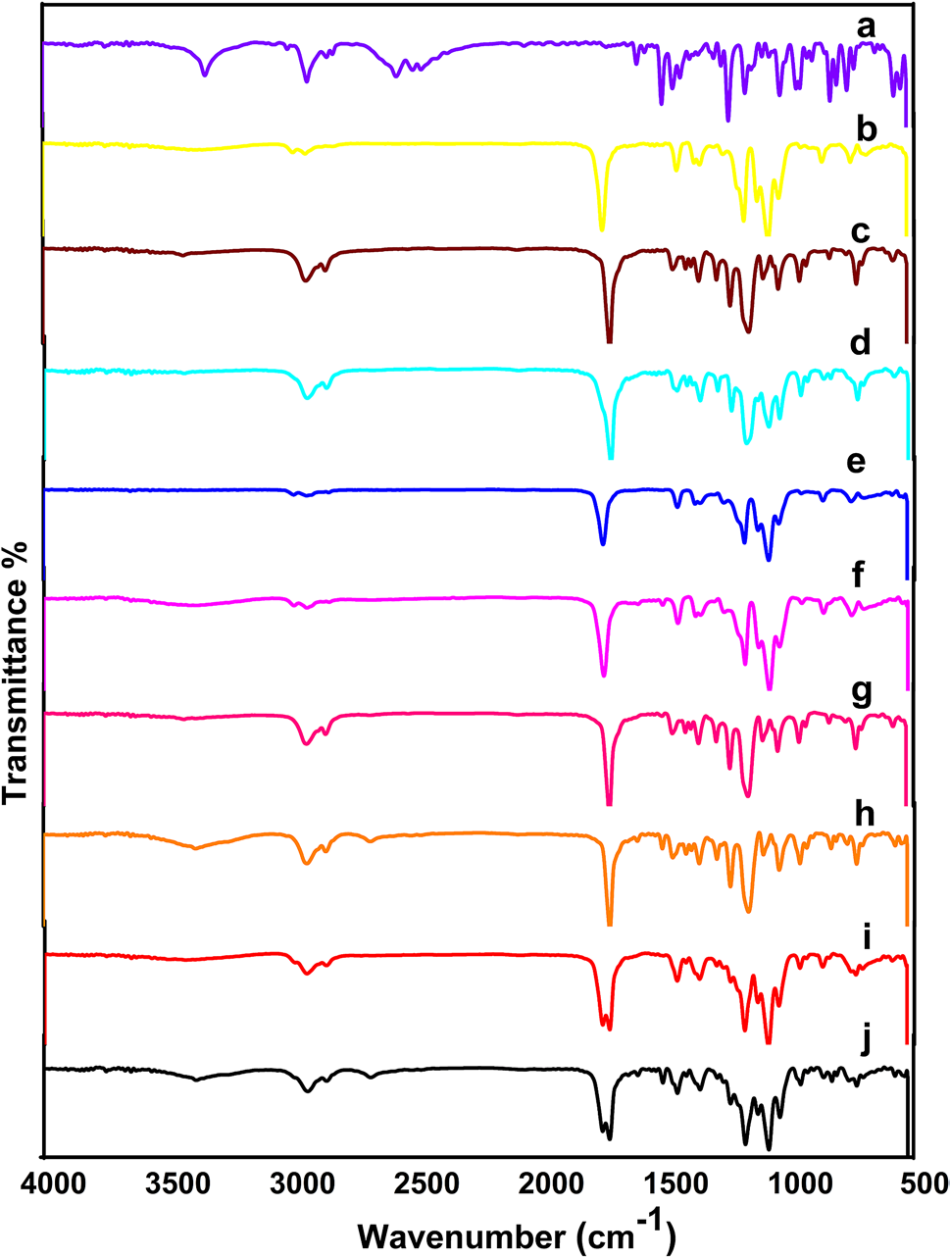
ATR-FTIR spectra of (**a**) VEN, (**b**) PLA cast film, (**c**) PCL cast film, (**d**) PLA/PCL blend cast film, (**e**) 10% PLA NFs (Plain), (**f**) VEN-10%PLA NFs (Medicated), (**g**) 14% PCL NFs (Plain) (**h**) VEN-14% PCL NFs (Medicated), (**i**) 10% PLA/PCL composite NFs (Plain), and (**j**) VEN-10%PLA/PCL composite NFs (Medicated).

In Fig. 2a, the spectrum of VEN has characteristic peaks at 3347 cm^− 1^ that are attributed to ‒O–H (stretch), 2937, 2858, and 2578 cm^− 1^ are attributed to –C–H (stretch); 1613 cm^− 1^ that exhibited from ‒C–C (stretch) and 1153 cm^− 1^ that is attributed to=C–N (stretch). In Fig. 2b, the PLA film spectrum shows the absorption peaks of the reduced –OH group at 3381 cm^− 1^, the C=O stretching of the carboxyl groups that were observed at 1749 cm^− 1^, and ether bond (C–O stretching) observed at 1183,1130, and 1086 cm^− 1^. Similar characteristic peaks are shown in Fig. 2c of the PCL film that has also exhibited a strong peak of C=O stretching at 1723 cm^− 1^ credited to the carboxyl groups of the PCL. As evident in Fig. 2e and g, there are no significant differences between the spectra of both PLA and PCL cast films and their plain NFs. The spectrum of the PLA/PCL blend (Fig. 2d) shows assignable peaks of both PLA and PCL, but no new peaks are noticed in its spectrum. A significant broad peak at 3347 cm^− 1^ was noticed (Fig. 2f, h, and j) that referred to O–H (stretch) of VEN. Meanwhile, the FTIR spectrum of F_3_ NFs (Fig. 2j), presented sharp absorption peaks at 1752 and 1726 cm^− 1^ of C=O stretching of the carboxyl groups of PLA and PCL, respectively. In comparison to the neat PLA and PLA-PCL blend; there was a band position shift from 1749 to 1752 cm^− 1^, corresponding to C=O stretching vibration. This shift describes the C=O group of the PLA involved in the interactions of the –OH group of PCL through hydrogen bonding as depicted in Fig. S2. Similar results were reported by Przybysz-Romatowska et al. ^40,41^. Additionally, drug-polymer systems may interact through hydrogen bonds leading to systems with much-improved drug loading, dissolution performance, and overall stability^42^.

#### Crystallinity evaluation of NFs

**Figure 3:**
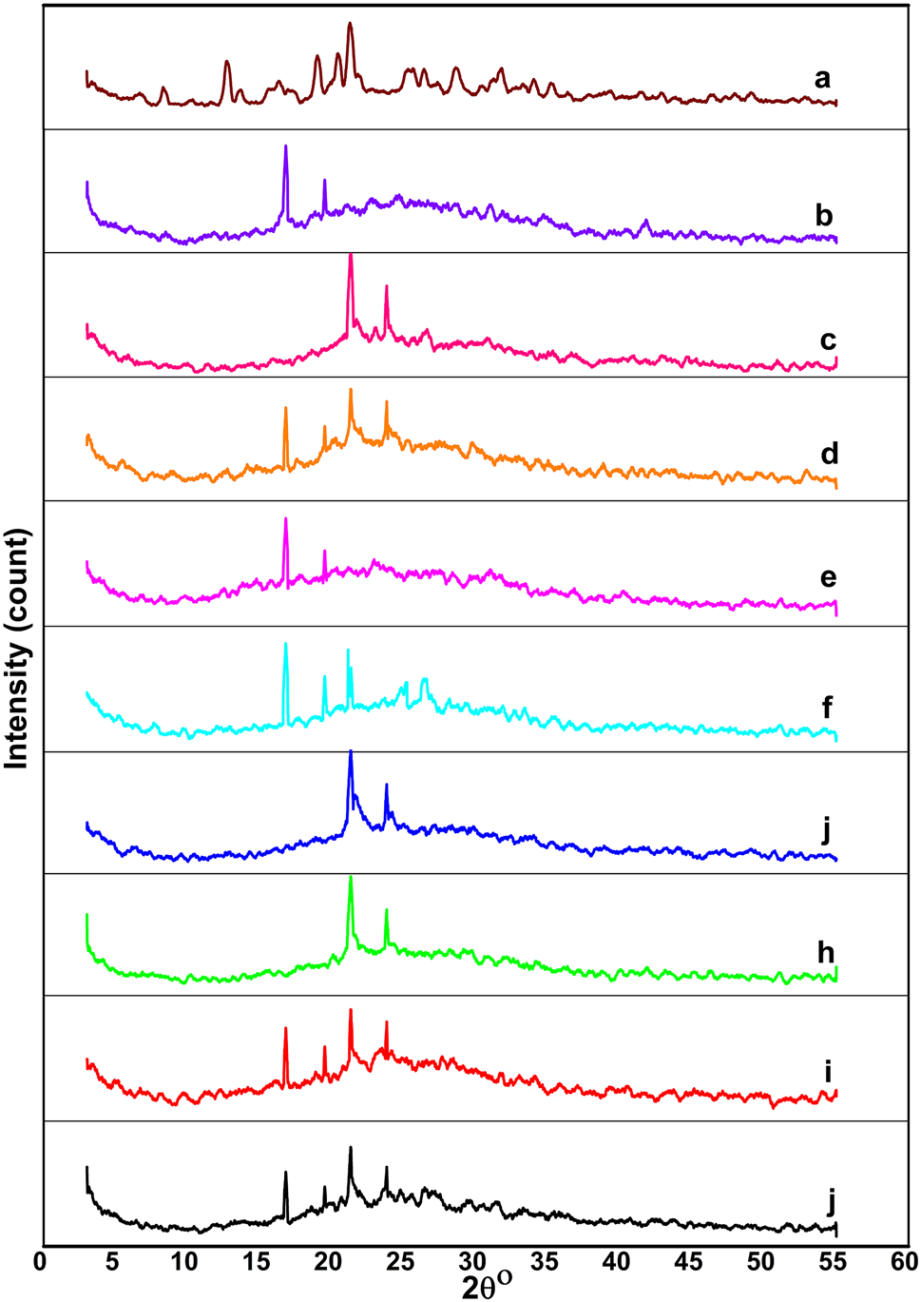
XRD patterns of (**a**) VEN, (**b**) PLA cast film, (**c**) PCL cast film, (**d**) PLA/PCL blend cast film, (**e**) 10% PLA NFs (Plain), (**f**) VEN-10%PLA NFs (Medicated), (**g**) 14% PCL NFs (Plain) (**h**) VEN-14% PCL NFs (Medicated), (**i**) PLA/PCL composite NFs (Plain), and (**j**) VEN-PLA/PCL composite NFs (Medicated).

XRD patterns of VEN, cast films, and NFs are evident in Fig. 3. As shown in Fig. 3a, VEN is a highly crystalline powder with characteristic reflections at 2θ of 6.7°, 8.3°, 12.7°, 13.6°, 16.4°, 18.9°, 20.3°, 21.3°, 21.5°, 25.1°, 28.6° and 35.1° (Bragg angles) well supported by the literature^43,44^. As seen in Fig. 3b, PLA cast film exhibits strong Bragg reflection that occurred at 2θ values of 16.8° and 19.5° and is ascribed to (200) (110) and (203) crystallographic planes of a-form PLA crystal, respectively. Meanwhile, the sharp diffraction peaks appeared at 21.3° and 23.8^°^, corresponding to (110) and (200) crystal plane diffraction of PCL, respectively (Fig. 3c). Figure 3d shows crystalline peaks of both PLA and PCL polymers in their blend. After electrospinning, typical amorphous structures of both F_1_ and F_2_ and their plains exhibited small intensities peaks at the similar positions of the PLA and PCL reflections.

#### Thermal analysis of NFs

**Figure 4:**
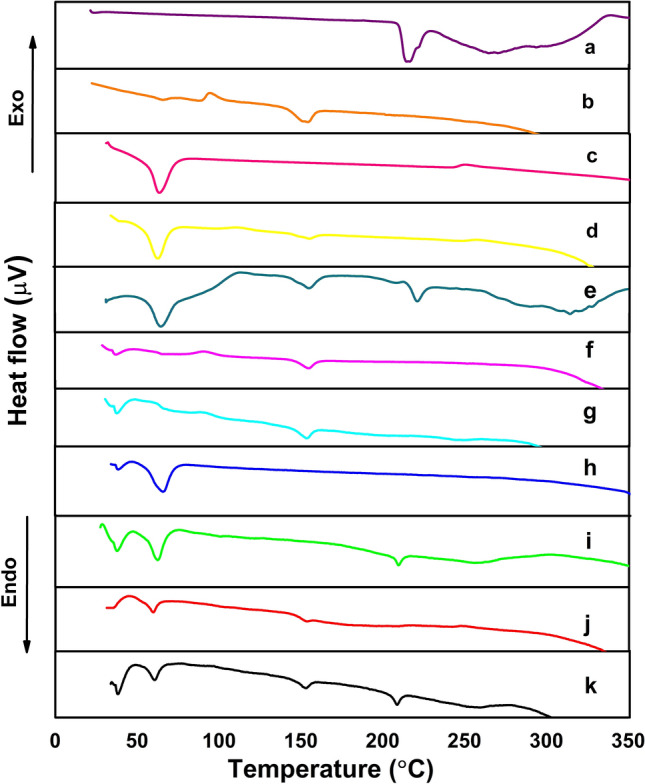
DSC thermograms of (**a**) VEN, (**b**) PLA cast film, (**c**) PCL cast film, (**d**) PLA/PCL blend cast film, (**e**) VEN-PLA/PCL cast film, (**f**) 10% PLA NFs (Plain), (**g**) VEN-10%PLA NFs (Medicated), (**h**) 14% PCL NFs (Plain) (**i**) VEN-14% PCL NFs (Medicated), (**j**)10% PLA/PCL composite NFs (Plain), and (**k**) 10% VEN-PLA/PCL composite NFs (Medicated).

DSC has been conducted to be a fast method for appraising physicochemical interactions between constituents of the formulation via the comparative studies of the thermal curves of pure substances, the polymeric NFs, and their drug-loaded fibers. Exemplary DSC heating thermograms are represented in Fig. 4. DSC thermogram of VEN, Fig. 4a, displayed a sharp melting endotherm at 211.87 °C exhibiting its crystalline nature^45^. Figure 4b showed a cast PLA film manifests both a glass transition temperature (T_gPLA_) at 65 °C and an endothermic melting peak (T_mPLA_) at 154 °C^46^. Whereas the cold crystallization exotherm exhibited a crystallization temperature (Tc) at 94 °C. About PCL, the melting process is located between 45 and 65 °C with a peak at 59 °C; Tg for PCL (T_gPCL_) cannot be seen in these DSC traces because the temperature program starts from room temperature, and the typical T_gPCL_ values are close to − 60 °C as shown in Fig. 4c^47,48^. DSC curves of PLA/PCL blends are very interesting for assessing the miscibility between these two components. As we can see in Fig. 4d and e, the DSC curve of PLA/PCL blends between 30 and 210 °C shows the same thermal transitions as the individual polymers. Meanwhile, the T_mPLA_ does not change with PCL addition, with values around 154 °C in their nanofibers (plain and medicated samples), as shown in Fig. 4f–k. All thermograms showed the absence of the solvents used.

#### Encapsulation efficiency of electrospun NFs

The entrapment efficiency is an indispensable criterion for evaluating the drug amount incorporated in NFs. All PLA, PCL, and PLA/PCL composite nanofibers have been delineated to have adequate encapsulation efficiency (≥ 95%).

#### In vitro drug release study

**Figure 5:**
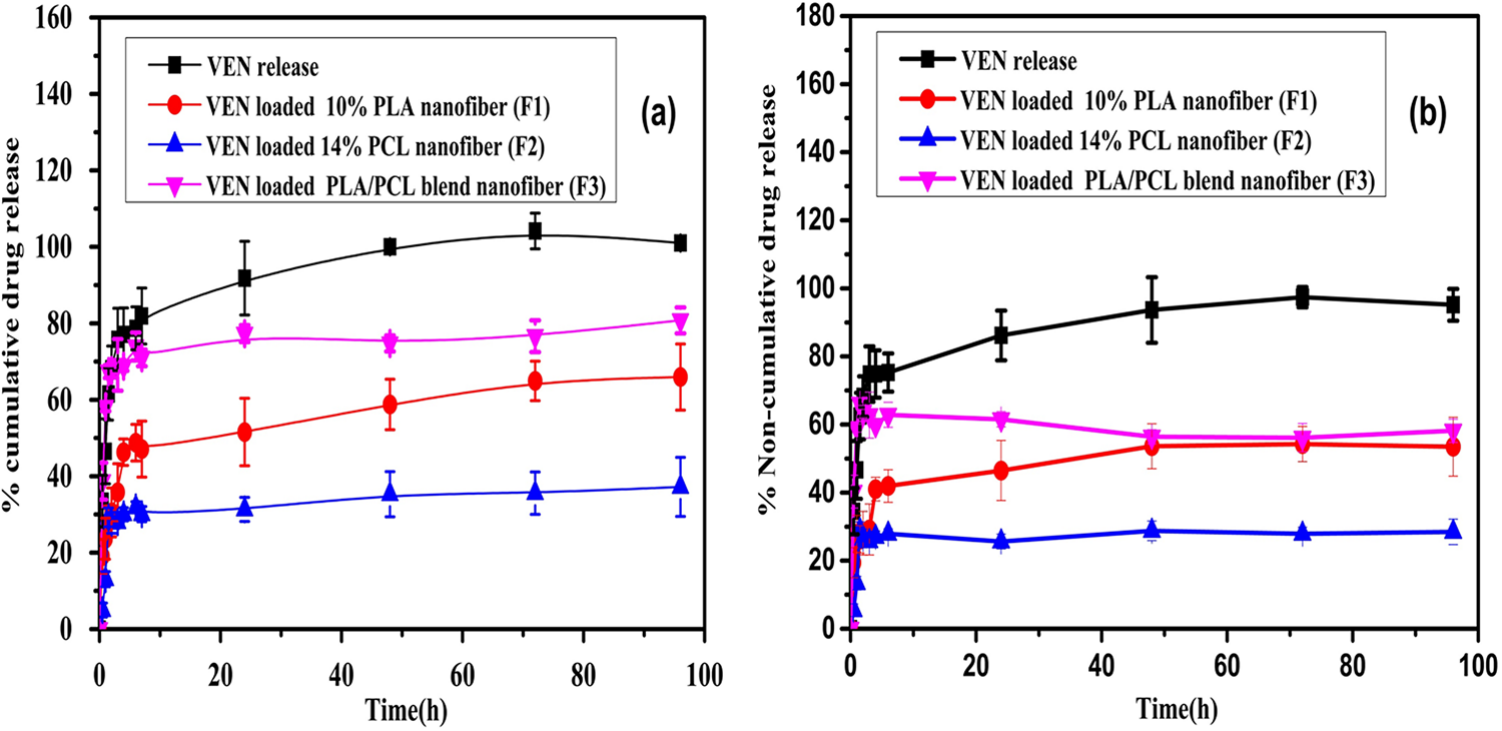
Drug release profiles of nanofibers for (**a**) cumulative, and (**b**) non-cumulative drug release amount in phosphate buffer (pH 7.4) at 37 °C and 100 rpm, data expressed as mean ± SD (n = 3).

The release study of the selected VEN-NFs was conducted in a phosphate buffer solution (PBS) at pH 7.4 for 96 h. Compared with the release profile of pure drugs under the same experimental conditions for cumulative and non-cumulative release amounts. As illustrated in Fig. 5a and b, all the drug-loaded NFs scaffolds have two stages (bimodal) of drug-release patterns. The pure drug displayed a rapid burst release (about 38% of the drug) in the initial 0.5 h followed by rapid release approaching 100% throughout seven h at pH 7.4 medium. The VEN-NFs showed a burst release (around 5%, 20%, and 30% from PCL, PLA, and PLA/PCL blended, respectively) during the initial stages of the test (the first 30 min), followed by a prolonged cumulative release pattern as shown in Fig. 5a. The maximum plateau of drug released was occurred within four days, which was 37 ± 7.7, 65 ± 8.6, and 80 ± 3.3% for PCL, PLA, PLA/PCL blended (F_1_) NFs, respectively. In comparison, F_3_ exhibited the highest release rate, which reached 80% over four days of the PLA and the PCL blended nanofibers. Figure 5b shows no major differences between both cases of cumulative and no-cumulative release behaviors.

#### Mathematical models for drug release kinetics

The selection of an acceptable model for relevant drug release data is chosen for defining the release characteristics using model-dependent slants. The initial “burst release” was considered for adjusted mathematical equations. The coefficient of determination values (R^2^) was calculated corresponding to four mathematical models. As illustrated in Table 2, the four formulations did not obey either zero-order or first-order kinetics. The best explanation of the release obeys Higuchi and Korsmeyer–Peppas models, as their plots showed high linearity^49^. For the implementation of the release mechanism explanation, the release mechanism is represented as a function of the diffusion exponent (n). VEN–release and F_1_ kinetic models at pH 7.4 fitted well with Fickian-diffusion (n < 0.5). However, both medicated F_2_ and F_3_ followed the non-Fickian diffusion (anomalous) model (0.5 < n < 1).

**Table 2:** Release kinetics of in vitro release data.

Model	Parameter	VEN	F1	F2	F3
Zero-order	R^2^	0.898	0.755	0.721	0.772
	K_o_	1.346	0.434	0.411	1.295
	Q_o_	0.372	0.371	0.121	0.536
First-order	R^2^	0.798	0.798	0.795	0.700
	K_1_	0.316	0.243	1.190	0.364
	Q_o_	1.354	0.755	0.133	1.416
Higuchi	R^2^	0.985	0.974	0.850	0.966
	K_H_	1.868	0.834	0.034	2.001
	Q_o_	0.057	0.109	1.108	0.085
Korsmeyer-Peppas	R^2^	0.951	0.995	0.991	0.986
	n	0.420	0.346	0.624	0.514
	K_KP_	0.179	0.241	1.430	0.263

Where; R^2^ is correlation coefficient; while, Ko, K_1_, K_H_ and K_KP_ are zero-order release, first order release, Higuchi, and Korsmeyer constants, respectively. Qo and n are the initial release amount (µg/mL) and diffusion or release exponent, respectively.

#### Ex vivo drug permeability through chicken pouch mucosa

**Figure 6:**
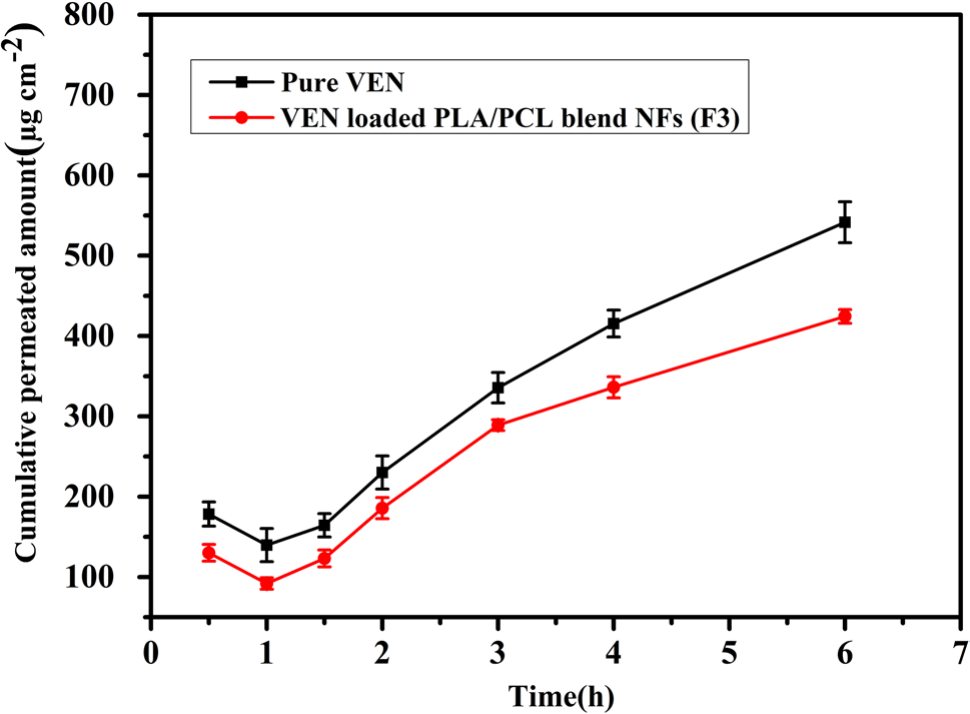
Ex vivo permeation profile of VEN solution and VEN-loaded PLA/PCL composite (F_3_) across chicken cyst mucosa into phosphate buffer (pH 7.4) at 37 °C, data stated as average ± SD (n = 3).

The chicken cyst is regarded as the best fit and convenient model mucosa for this study; it is widely available and presents an alternative to partially keratinized rabbits’ mucosa and the keratinized mucosa of hamsters/ rats. Otherwise, dogs, monkeys, and pigs are also considered non-keratinized mucosa, but they have been restricted in this study to have more superfine and highly porous mucosa than that of humans^36^. On comparing F_3_ with the control VEN solution, the rate of permeation through the NFs system significantly decreased (ρ < 0.0001) relative to the corresponding drug solution from 76.348 ± 1.04 to 56.812 ± 1.02 µg h^-1^, respectively. Figure 6 displays that, at six h, the quantity of VEN infiltrated from the drug solution exceeds that of the nanofiber system. Regarding permeation flux (J), which is defined as the drug amount infiltrated per centimeter square per hour^50^, the composite nanofiber decreased the flux of VEN significantly from 22.063 ± 0.8 to 16.419 ± 0.5 µg cm^− 2^ h^− 1^ (ρ = 0.0005). Another parameter of permeation coefficient (P) used to measure the movement rates of drug molecules across a convoluted polymer was also determined. Data in Table 3 displayed that the permeation coefficient of F_3_ was diminished compared to the control; however, it was not significantly different (ρ = 0.0653).

**Table 3:** Ex vivo permeation parameters of VEN solution and VEN-loaded PLA/PCL composite NFs (F3) through chicken cyst mucosa determined at 37 °C, data defined as average ± SD (n = 3).

Parameter formulation	Rate of permeation (dQ/dt)	Permeation flux (J) (µg cm^−2^ h^−1^)	Permeation coefficient (P) (cm h^-1^)
Pure VEN	76.348 ± 1.04	22.063 ± 0.8	0.011 ± 0.002
F3	56.812 ± 1.02	16.419 ± 0.5	0.008 ± 0.0005
Unpaired t-test	(ρ < 0.0001)	(ρ = 0.0005)	(ρ = 0.0653)

ρ value that represents the outcome of the unpaired t-test analysis (at the level of ρ < 0.05).

**Table 4:** IC_50_ of VEN, plain, and combined samples in the studied cell lines after 48 h.

IC_50_ (µg/ml)	Formula		
	Vero	Huh-7	OEC
VEN	182.74 ± 17.49	229.88 ± 22.27	217.55 ± 10.96
Plain	993.15 ± 67.95	596.08 ± 34.26	440.48 ± 60.52
F3	498.58 ± 61.35	337.04 ± 45.07	250.62 ± 11.90

IC_50_; the concentration that killed 50% of cells, Vero; Green monkey kidney, Huh-7; Liver cancer, OEC; Oral epithelial cell (mean ± SD; n = 3).

#### Histological examination

**Figure 7:**
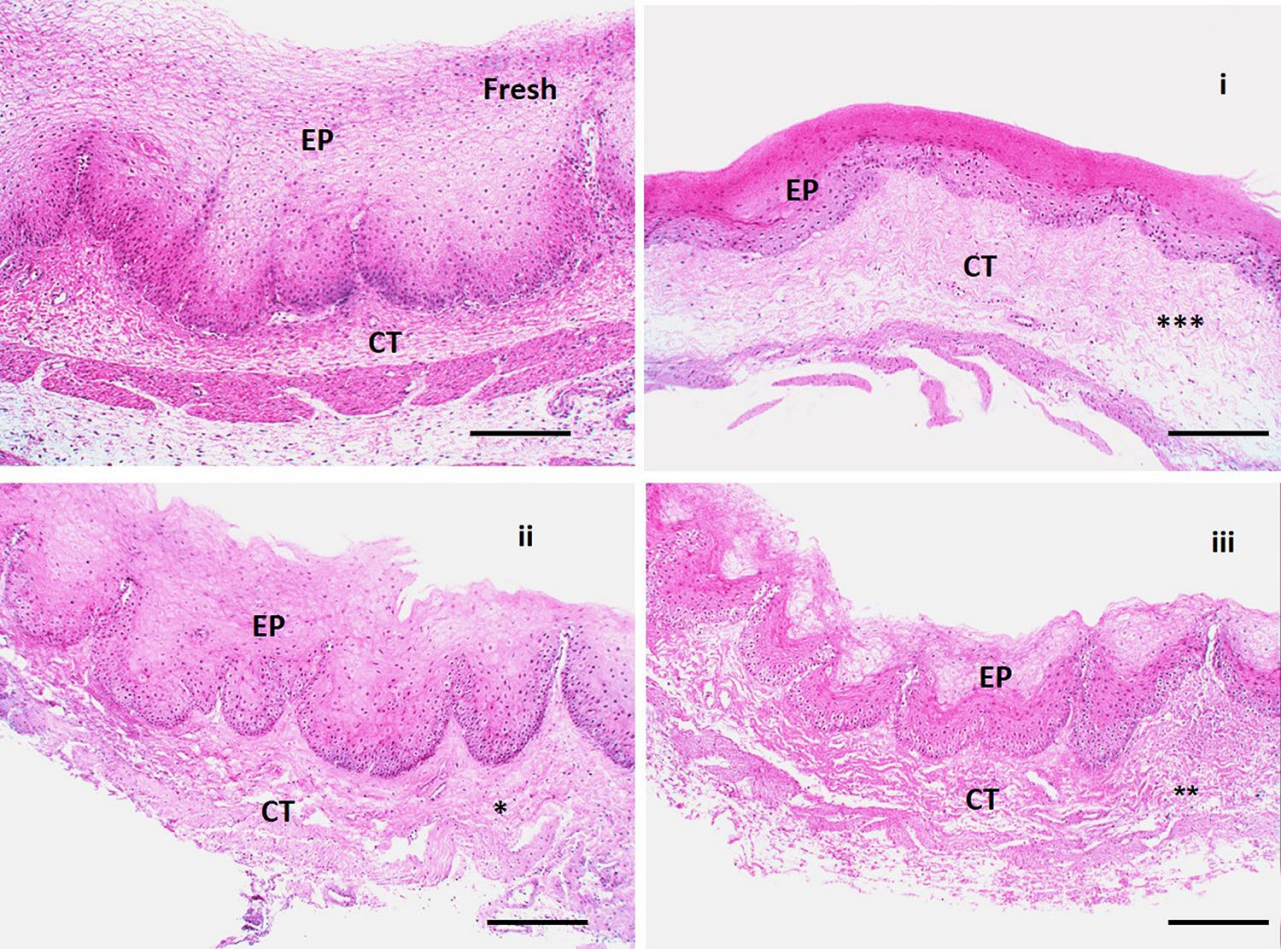
Histopathological pictures of chicken cysts mucosa utilized in ex vivo permeation for a fresh sample as control mucosa, **(i)** mucosa exposure to VEN solution, (**ii)** mucosa exposure to plain NFs, and **(iii)** mucosa exposure to medicated NFs (F_3_) using H&E stain X:100 bar 100. (*ρ < 0.05, ** ρ < 0.01, ***ρ < 0.001 vs corresponding value of fresh group).

**Figure 8:**
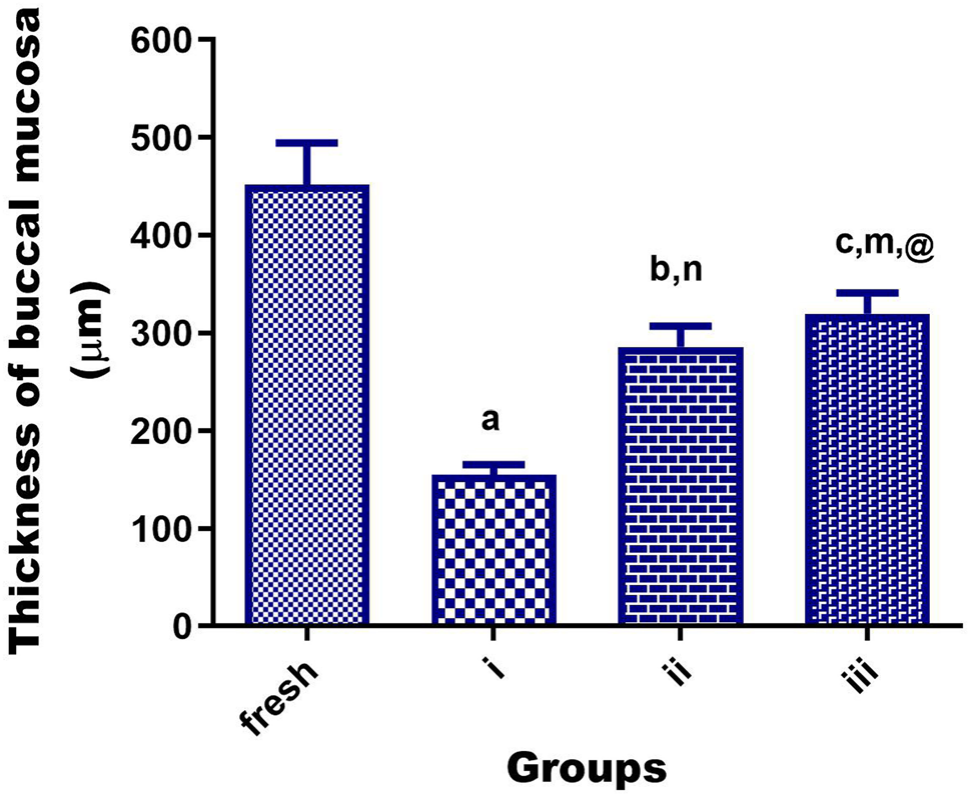
Statistical analysis of the thickness of H&E-stained sections of chicken cyst tissues (as buccal mucosa model) using one-way analysis of variance (ANOVA) followed by the Tukey's for individual comparison of group means to show a significant reduction in fresh mucosa group when exposed to a pure drug of VEN group **(i)**, plain NFs group **(ii)** or medicated NFs (F_3_) group **(iii)**. **Notes:** Different small alphabetical letters mean significant when ρ < 0.05. (^a^*ρ* < 0.0001; ^b^*ρ* < 0.001; ^c^*ρ* < 0.01 versus corresponding value of a fresh group, ^n^ *ρ* < 0.01 versus corresponding value of VEN group, ^m^*ρ* < 0.05 versus corresponding value of VEN group, ^@^*ρ* > 0.05 versus corresponding value of plain group).

Figure 7 represented four H&E-stained sections of the mucosa viz., fresh (control), positive control-treated (VEN), negative control-treated (plain), and mucosa treated with VEN NFs (F_3_). The microscopic pictures of the fresh group manifest normal non-keratinized stratified squamous epithelial lining (EP) resting on folded basement membrane with normal lamina propria underneath composed of highly vascular connective tissue (CT) with no signs of cellular injury, degeneration, or inflammation. However, as shown in (Fig. 7i and Fig. 8i), the mucosa treated with VEN reveals a marked reduction in mucosal thickness and extensive damage to the underlying CT (ρ < 0.0001). Negative control (plain, Fig. 7ii and Fig. 8ii) treated mucosa was found to be intact with preserved structure and a slight reduction in mucosal thickness and loosening of the underlying CT (ρ < 0.001). After treating mucosa with VEN composite NFs (F_3_, Fig. 7iii, and Fig. 8iii), neither cell necrosis nor structural damage was noticed.

#### In vitro cytotoxicity study

A WST-1 assay was applied to study in vitro cell viability as an indicator of cell damage or cytotoxicity. The cytotoxic effects of VEN, plain NFs, and medicated NFs (F_3_) were evaluated in three different cell lines, e.g., Huh-7, OEC, and Vero cells.

According to these preliminary toxicity results (Table 4), the WST-1 assay judged that F_3_ is non-toxic and cytocompatible compared to the free VEN as exhibited values of IC_50_ indicating too high viability of the exposure cells up to 2 days. The percentage of cell viability for all treatments remained above 80%, with no statistical difference between fibrous mats with and without VEN or between negative control (Pure VEN). It reflects that neither polymers nor the amount of drug released are cytotoxic, falling within the accepted limit analyses^51^.

## Discussion

Electrospun NFs have received special attention as drug delivery systems owing to their distinct functional features and easy fabrication techniques^15^. The biocompatible polymeric materials are biodegradable poly (α-hydroxy ester) based polymer families like poly (lactic acid) and poly(ε-caprolactone), as shown in Fig. S1. This work focuses on developing novel electrospun polymeric NFs for buccal delivery of VEN to avoid the hepatic metabolism and enzymatic degradation in the GIT and develop an effective controlled drug release. VEN-loaded NFs were successively fabricated by the electrospinning method using biocompatible polymers. All of the parameters involved in the electrospinning process were carefully optimized, such as polymer concentration, polymer ratio in the case of composite fibers, flow rate, applied voltage, and tip-to-collection distance. In the present study, the polymer solution (PLA, PCL, and PLA/PCL blend) and VEN in their respective solvents were electrospun under the applied electrical potential of 20 kV over a tip-to-collector distance of 15 cm and flow rates of 1.0, 0.6, and 0.3 mL/h, respectively.

The morphological studies and size of the cast films and NFs were evaluated by SEM, as seen in Fig. 1. The smooth fracture surface of the cast film of PLA and PCL is attributed to its relatively brittle fracture manner in the two films before the electrospinning process. While electrospinning of 10% PLA yielded optimum fibers with an average diameter of 400–500 nm under well-defined conditions, their morphology may be due to the selected binary solvent system of CHM: DMF (80:20) that has low surface tension, suitable viscosity, high miscibility, and high conductivity which aid in the production of nanofibers^52^. Similarly, the boiling point of each solvent in the binary system plays a significant role in NFs formation. DMF has a higher boiling point than CHM, which offers enough time for the spinning process to be completed correctly without any bead formation^53^. As the increment of PCL concentration rose from 10 to 14%, there was a gradual increase in the fiber diameter. This observation indicated that increased PCL concentration increases the electrical force exerted on the jet, increasing the mass throughput. That results in chain entanglement, consequently growing the average diameter size of NFs^54–56^. PCL nanofibers appeared with smaller diameters than PLA NFs due to the virgin polymer (PCL) having a higher degree of molecular orientation, crystallinity, and mechanical properties^57^.

Representative SEM images of plain and medicated NFs revealed smooth and regular NFs with no drug crystals identified on/or outside the surface.

The film with the blended fiber at a PLA/PCL ratio of 80:20 is characterized by the smoothest texture and the highest orientation. These observations confirm the high compatibility of the drug with a polymer–solvent matrix that belongs to a homogeneously distributed drug within the fiber preparation. The addition of VEN to the polymer solutions has no significant effect on the viscosity of the polymeric solutions or the average diameter size of NFs due to its high solubility in the polymeric solution. We focused on the PLA/PCL binary system to obtain a narrow and smooth diameter of VEN-NFs.

As can be confirmed in Fig. 2, FTIR spectra demonstrated that the assignable peaks of the drug are visible in all the drug-loaded NFs, in addition to the lack of novel absorption peaks. Fig. S2. illustrates the proposed interactions between biopolymers (PLA and PCL) and the drug molecules. These outcomes confirm that the hydrogen bond occurs between the drug and the utilized polymers during the preparation of NFs.

According to the XRD results in Fig. 3, PLA was less pronounced than PCL because of its lower crystal absorption density and showed a more pronounced amorphous scattering. It may be related to the different degrees of molecular deformation during the electrospinning procedure^17^. The XRD patterns of the prepared NFs may be inferred to the less perfect crystalline structure indicating wide amorphous scattering peaks. During the electrospinning process, the fast solidification of the fibers restricts the order of the three-dimensional polymer lattice and retards the crystallization process^58^. Therefore, it can be said that VEN was successfully loaded in composite NFs. These results are in agreement with the literature^17,26,59^.

DSC thermograms (Fig. 4) confirm the semi-crystalline nature of the PLA polymer. PCL accelerates the crystallization rate of PLA with a small effect on its final crystallinity degree^46,60^. These PCL crystals designed in the boundaries may supply the nucleating sites for PLA to crystallize. An overall decrease was revealed in the crystallinity of both PCL and PLA configuration in NFs composite in correlation with XRD analysis^60^. Of these, blending PLA and PCL overcame the shortcomings of both PLA and PCL^61,62^.

The heat flow value of VEN in the blended biopolymers decreased, indicating that the drug-loaded fibers samples contain free amorphous regions. It may be attributed to the extremely rapid vaporization of the solvent from the NFs during the electrospinning process, leading to the failure of the drug molecules to form a complete crystalline lattice within the NFs^63^. DSC studies confirmed that the drug molecules were evenly distributed at the molecular level in the nanofibers matrix and were existent in an amorphous state, favoring the good compatibility and stability of the composite NFs^63^. The drug, VEN, is not influencing the melting and glass transition temperatures, which agrees with FTIR and XRD spectra.

Even though VEN had more water solubility (534 mg/mL) than other water-soluble drugs used in studies^27^, all prepared nanofibers (F_1_, F_2_, and F_3_) revealed an immense encapsulation efficiency percentage. It may be owing to the exceptional high surface area of the NFs and the absence of drug loss during NFs preparation^64^.

In vitro release study of VEN from nanofibers represents a sustained/controlled drug release as the drug disperses to the release medium across the carrier^39^. The cumulative amount of the drug released demonstrated that the quick drug release from the buffer solution might be ascribed to the high solubility of VEN in water and its availability to contact directly with the diffusion cellulose membrane^65^. Prior studies had evidenced that the initial release occurred because the hydrophilic drugs were readily available on the surface of NFs when implanted in hydrophobic polymers^39^, as demonstrated by microscopic analysis^39,51^. The amount of drug release from the proposed formulation reached about 80% per four days compared with the literature, such as 56%/10 h for venlafaxine hydrochloride-loaded chitosan nanoparticles^6^, 89.97%/5 h for PVA/CMC nanofiber^8^, and montmorillonite-PLGA nanocomposites that reached to 100%/12 h^26^.

In comparison, it could be suggested that the presence of the polymers allowed migration of the entrapped drug from NFs through a longer path, resulting in less burst effect and controlled release behavior as compared to pure VEN, as shown in Fig. 5.

PCL NFs revealed the lowest release rate attributed to the partial crystallinity of PCL and the presence of five hydrophobic -CH_2_ moieties in its repeating units^66^. Due to the hydrophobicity and regarding the drug-polymer hydrophilic-hydrophobic interactions, the interaction of VEN with the hydrophobic segment of the biopolymer (PCL) is stronger, resulting in a slow-release^67,68^. PLA NFs showed a higher cumulative release rate that was attributable to the high amorphous nature of PLA. The degradation of PCL does not create acidic byproducts, unlike the degradation of PLA. It makes PCL a more adequate nanomaterial for improving long-term implantable devices due to its sluggish degradation rate^69^. F_3_ drug release profile confirmed the enhancement of the blended features due to the presence of both polymers in the same matrix. These explanations affirmed that the NFs might be able to effectively and sustainably deliver the drug, thereby enhancing patient compliance. Some authors proposed the use of fibrous mats can deliver the drug across the skin via transdermal delivery, providing the therapeutic concentration within 24 h and sustaining its delivery for several days^51^. Generally, the drug release from NFs mats was modulated by a mixed mechanism of drug diffusion from nanopores of the NFs and degradation of the polymeric matrix^39^. There are insufficient physical and chemical interactions between the hydrophilic drug molecules and the hydrophobic polymer matrix, as evident in the ATR- FTIR spectra study.

The release mechanism of VEN and F_1_ is fitted well with the Fickian mechanism, indicating that the release of the drug is mainly mediated by its diffusion across the polymeric matrix^70^. While the release of F_2_ and F_3_ followed an anomalous non-Fickian distribution, signified that the dismissal was controlled by drug diffusion and polymer erosion^71^. It consists of two dominant driving forces: drug desorption and diffusion-controlled release kinetics^39^. Compared to the published works for VEN release kinetic models and mechanisms (Table S1) that agree with the behavior of the release of VEN from PLA/PCL blended NFs^8,11,13^. The combined outcomes conclusively demonstrate that F_3_ is a good delivery system of VEN and should be studied further.

The ex vivo results emphasize the enhancement of the sustained drug release and the noticeable decrease in the permeability of the drug from NFs (ρ < 0.05) compared to the dug solution, as seen in Fig. 6. VEN is a BCS class I drug that is characterized as having a rapid absorption from the GIT leading to a short duration of action^72^. These results indicate that the prepared NF formulation could be a facile and green approach for buccal administration with minimal drug permeation, reduced systemic absorption, and enhanced patient convenience.

Buccal histopathology is useful for studying the toxicity effects of the selected formulation on the integrity of buccal mucosa, as illustrated in Fig. 7 and 8. The exhibited observations are ascribed to the pH value of VEN NFs (6.58 ± 0.32), which was within the pH range of human buccal mucosa (6 –7.5), reflecting its safety for buccal administration^71^. Additionally, it may be related to the good biocompatibility, favorable mechanical properties, low immunogenic reactions, and chemical versatility of the utilized alloplastic (PLA and PCL) materials^73^.

Cytotoxicity tests have been considered the first step in identifying biosafety testing of active compounds. It is necessary to confirm that these fibrous mats are safe to be used in contact with the skin without being toxic to cells. In vitro cytotoxicity study confirmed that the nanocomposite is safe and cytocompatible as electrospun NFs. These may be due to its biocompatible composition and the mild formulation procedures undertaken. A medical device is considered non-cytotoxic when cell viability is equal to or superior to 70%, according to ISO 10,993–5, 2009. In this sense, the results demonstrate that electrospinning's VEN-loaded PLA/ PCL delivery systems are potentially safe for buccal applications^51^.

## Conclusion

Electrospinning is a highly sophisticated, robust, and applicable technique to fabricate ultrafine fibers from different electrostatic fluids. The optimized formula was successfully fabricated by blending PLA and PCL at a ratio of (1:1) fixed at 10% w/v. Morphological analysis refers to free-beads, smooth, very narrow, and uniform electrospun fibrous structures of PLA, PCL, and PLA/PCL NFs mats. Spectral and thermal patterns are matched with the drug release profiles that affirm the PLA is more amorphous than PCL and manifests an enhanced sustained release profile with a decline in the permeability of the drug from the new blend at pH 7.4. Also, highly fitting models suggest the drug release mechanism is close to Higuchi and Korsmeyer–Peppas models. Histopathology and cytotoxicity studies showed no noticeable histological effects, confirming the safety of buccal VEN delivery. In a nutshell, PLA/PCL blend NFs may be deemed a prospecting and safe controlled-release strategy for buccal delivery of antidepressant drugs to lessen the drug administration frequency and the occurrence of side effects to increase the effectiveness of the drug and thereby improve patient compliance. To make a complete judgment about the developed nanosystem, further studies, including experimental animals, are beyond the scope of this paper and will be investigated in our future work.

## Supplementary Information

Supplementary Information.

## Data Availability

The data that support the findings of this study are available from the corresponding authors on request.
