# Supplementary material for: Fabrication and characterization of electrospun nanofibers using biocompatible polymers for the sustained release of venlafaxine
Source: Sci Rep. 2022 Oct 27;12:18037. doi: 10.1038/s41598-022-22878-7 (PMC9614003; doi:10.1038/s41598-022-22878-7)
Supplement: Supplementary file 1 — Supplementary Information. [file 41598_2022_22878_MOESM1_ESM.docx]

**Table S1:** Comparison between the assessment results of different formulations of Venlafaxine nanoparticles.

| **Formulation** | **Encapsulation efficiency %** | **Cumulative amount %/h** | **Kinetic model** | **Ref.** |
| --- | --- | --- | --- | --- |
| Venlafaxine Hydrochloride-Loaded Chitosan Nanoparticles | 70 | 56 /10 | - | ^6^ |
| PVA/CMC nanofiber | - | 89 /5 | Higuchi | ^8^ |
| Wax matrices and double-layer coatings | 40 | 90 /12 | Higuchi | ^11^ |
| Dendrimer–venlafaxine | - | 50 /6 | - | ^12^ |
| Ethyl cellulose/Eudragit to HPMC Hydrogel | - | 90 /16 | Higuchi | ^13^ |
| Venlafaxine-nanoparticles | 76 | 64 /24 | - | ^25^ |
| Montmorillonite-PLGA nanocomposites | 88 | 100 /12 | - | ^26^ |
| Polyester dendrimer | - | 40 /6 | - | ^27^ |
| PLA/PCL nanofibers | ≥95 | 80 /96 | Higuchi | **This work** |

**Figure S1.** The chemical structures of a) Venlafaxine hydrochloride and b) PLA, and c) PCL.

**Figure S2.** Proposed schematic mechanism of the interaction of PLA and PCL biopolymers loaded VEN molecules.
